# Isolated Renal Laceration on Point-of-care Ultrasound

**DOI:** 10.7759/cureus.2113

**Published:** 2018-01-25

**Authors:** Madeline M Grade, Cori Poffenberger, Viveta Lobo

**Affiliations:** 1 Department of Emergency Medicine, Stanford University School of Medicine

**Keywords:** point-of-care ultrasound, trauma, fast exam, emergency medicine, renal inury, hematuria

## Abstract

We report a renal laceration identified on a point-of-care ultrasound (POCUS) performed in the emergency department on a 58-year-old female presenting after blunt trauma. Emergency workup demonstrated a right flank abrasion with tenderness to palpation, hematuria, and decreasing hematocrit. A Focused Assessment with Sonography in Trauma (FAST) exam, performed as part of the intake trauma protocol, identified positive intraperitoneal fluid in the right upper quadrant. A computed tomography (CT) scan established a diagnosis of isolated right renal hematoma arising from a Grade IV laceration, with no collecting duct involvement. This report reviews the sonographic distinction between a renal hematoma and a positive FAST exam, and emphasizes the vital role ultrasound plays in the evaluation of the trauma patient.

## Introduction

Renal injury occurs in 1%-5% of all traumas [1]. In the setting of a suspected renal trauma, point-of-care ultrasonography can play an important role in recognizing the evidence of injury and aiding in triage. Patients are broadly divided into three groups: those with hemodynamic instability, who may require surgical exploration if stabilization cannot be obtained for a computed tomography (CT) scan; those with hemodynamic stability and hematuria, who are typically evaluated with a CT scan; and those with hemodynamic stability, no hematuria, and negative point-of-care ultrasound (POCUS), who may be managed with clinical observation [2]. Kidney injury severity is described using Grades I-V on the American Association for the Surgery of Trauma scale, a validated predictor of clinical outcome [3], depending on the degree of anatomic involvement. The primary indicators of renal trauma on ultrasound are subcapsular hematoma, perinephric hematoma, or calyceal dilation associated with internal echogenicity [4]. Standard B-mode sonography has low sensitivity but high specificity for such findings [5-6], and the subtleties between a positive Focused Assessment with Sonography in Trauma (FAST) exam and renal hematoma are infrequently reviewed.

## Case presentation

A 58-year-old female with a history of hypertension and open cholecystectomy sustained a ground-level fall while jumping from one stump to another. After initially visiting her local urgent care, she was transferred to the emergency department on a minor trauma protocol. She endorsed flank pain and hematuria, but no dizziness or pain in other locations and the remainder of her review of systems was negative. She arrived hemodynamically stable, with initial vitals demonstrating a blood pressure of 148/92 mmHg, heart rate of 79 beats/min, respiratory rate of 12 breaths/min, oxygen saturation of 98% on room air, and temperature of 37.1°C (98.7°F). She was alert, oriented, and answered all questions appropriately. Her first and secondary trauma surveys revealed a right flank abrasion as well as tenderness to palpation in the right upper quadrant and at the right costovertebral angle. The remainder of her physical exam was unremarkable.

**Figure 1:**
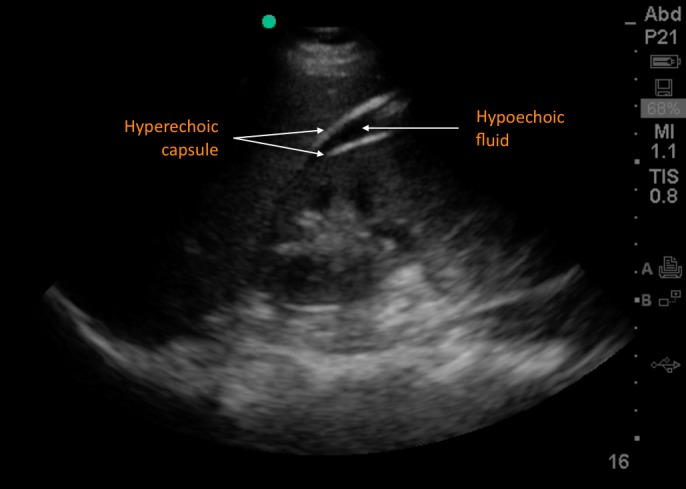
Point-of-care ultrasound visualization of the renal hematoma Right upper quadrant view demonstrates hypoechoic and heterogenous fluid between the liver and right kidney, bordered by hyperechoic capsular tissue, consistent with a subcapsular perirenal hematoma.

In tandem with the secondary survey, a FAST was conducted using a 5-1 MHz phased array probe (Sonosite, Bothell, WA) to evaluate for possible intraperitoneal or pericardial bleeding. The assessment was negative in the subxyphoid, left upper quadrant, and pelvic views, but positive for fluid in the right upper quadrant (Figure 1). The fluid was identified in what was initially interpreted as the hepatorenal recess, commonly referred to as Morison’s pouch, with some hyperechoic blood clotting. Upon closer examination, the bright ribboning of Gerota’s fascia surrounding the kidney was visible on both sides of the fluid collection, suggesting an alternate scenario of subcapsular fluid originating from injury to the kidney itself.

**Figure 2:**
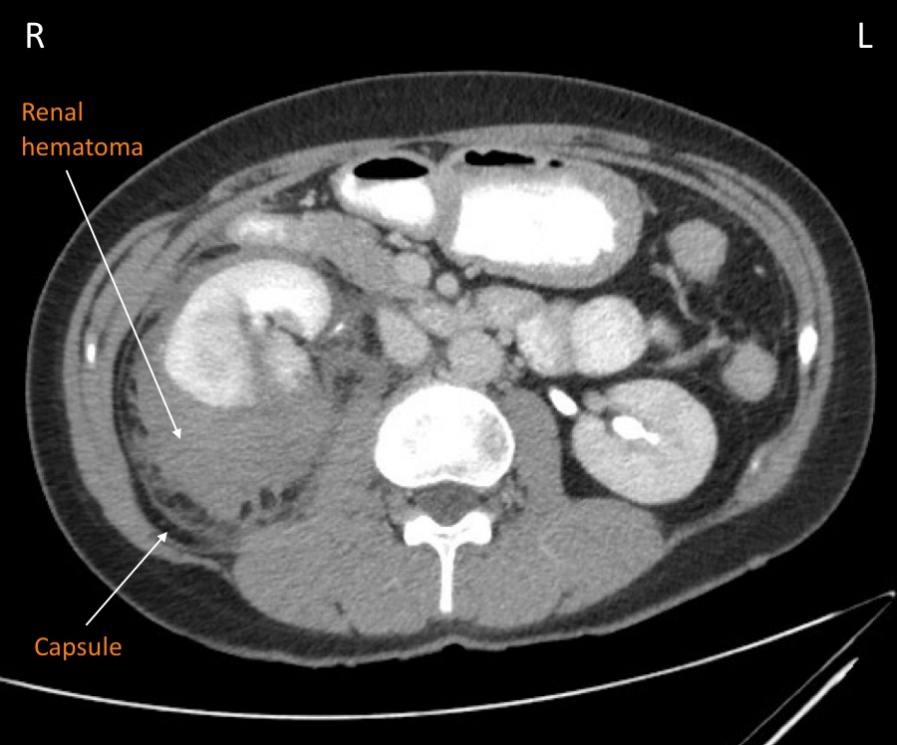
Computed tomography visualization of renal hematoma Coronal view of the abdomen demonstrates Grade IV kidney laceration and large subcapsular hematoma.

A CT scan was obtained, demonstrating Grade IV laceration of the inferior pole of the right kidney extending to the renal hilum (Figure 2). There was no evidence of active hemorrhage, no extraluminal urine to suggest damage to the collecting system, and no involvement of the renal vasculature. A large retroperitoneal, subcapsular perirenal hematoma was observed extending from the superior pole of the right kidney to just beyond the bifurcation of the left common iliac arteries, entirely contained within Gerota’s fascia. A urinalysis exhibited full field red blood cells. The laboratory findings included a white blood count of 18.8, initial hematocrit of 35.9%, and a normal creatinine at 0.81. Subsequent hematocrit measurements were 33.1% and 30.7%.

Both the general surgery and urology services provided consultation on this patient, recommending conservative management in light of the isolated injury and hemodynamic stability. The patient was admitted, placed on bedrest, and continued on intravenous fluids. She was followed with serial examinations and six-hour complete blood counts. Over the course of her stay, her hematocrit stabilized at 30.6%. Her gross hematuria resolved by the following morning, after which she could ambulate without difficulty, and she was discharged home with a plan to follow up in the urology clinic. Follow-up ultrasound imaging showed interval reduction in the hematoma surrounding the right kidney, down to approximately 12.84 x 9.2 x 5.4 cm. A final follow-up CT demonstrated organization and loculation of a 5.0 x 5.4 x 5.8 cm heterogeneous right perinephric collection with a peripheral rim of hyperdensity, on the whole consistent with a hematoma decreasing in size over time.

## Discussion

**Figure 3:**
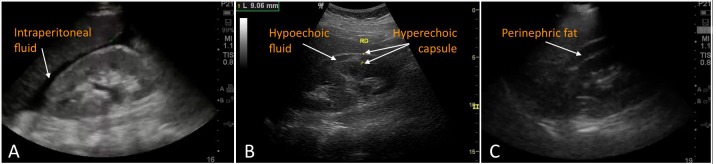
Ultrasound comparison of positive FAST, renal hematoma, and common artifact 3A demonstrates a classic positive Focused Assessment with Sonography in Trauma (FAST) exam, indicating free intraperitoneal fluid resulting from blunt abdominal trauma (courtesy of Dr. Viveta Lobo). 3B depicts another example of subcapsular perirenal hematoma, which arose after extracorporeal shock wave lithotripsy (courtesy of Dr. Bruno Di Muzio, Radiopaedia.org, rID: 30289). 3C illustrates the ‘double line sign’ artifact, which can be mistaken for a positive FAST (courtesy of Dr. Viveta Lobo).

Trauma patients frequently present with multiple injuries, and significant bleeding can occur without obvious changes in vital signs. Skillful use of POCUS can greatly aid in the rapid identification of intraperitoneal bleeding. When conducting a FAST, the right upper quadrant is the most sensitive location for free fluid in the supine patient, specifically in the gravity-dependent region of the superior paracolic gutter around the caudal liver edge (Figure 3A) [7]. A proper view must include the liver through its caudal tip, typically directly adjacent to the kidney, which is demarcated by its distinctly echogenic fibrous capsule and some amount of perinephric fat.

In a renal injury, one might encounter a range of sonographic findings [4]. The classic presentation is a hematoma located in the subcapsular or perinephric regions as seen in Figure 3B, though kidney fracture can also result in parenchymal hematoma and calyceal dilation. Organ hematomas can appear heterogenous and echogenic in the acute phase, becoming more hypoechoic relative to the surrounding tissue over time. If more advanced sonographic modalities such as color or power Doppler are available, an absence of perfusion can be detected in the damaged portion of the kidney. Bowel gas and lack of solid organ interface can make visualization of fluid in the anterior pararenal space difficult, while fluid in the posterior pararenal space must be distinguished from intraperitoneal fluid in the Morison’s pouch.

A more familiar explanation for the finding of two hyperechoic lines on either side of an apparent fluid collection between the liver and kidney is, in fact, an artifact; the “double line sign” arises due to hyperechogenicity of perinephric fat collections and can quite commonly be mistaken for a positive FAST (Figure 3C) [8]. This normal anatomical structure can be distinguished from a true fluid with careful observation. Similar to a pericardial fat pad surrounding the heart, its hypoechoic region may contain low-level echoes, and the perinephric fat moves with the kidney during respiration.

In this case, the appropriate management of the patient’s isolated renal laceration in the setting of hemodynamic stability was a clinical observation, consistent with the majority of renal trauma cases [1]. Given her hematuria, in addition to the evidence of a renal injury on ultrasound, a CT was indicated to assess the extent of damage and the possible involvement of the vasculature or the collecting system [2]. With its relatively low sensitivity, a normal ultrasound should not outweigh legitimate clinical suspicion in the decision to obtain CT imaging. However, in the absence of hematuria, ultrasound could conceivably aid in preventing unnecessary CT scans in low-level trauma scenarios [9]. In addition, the ability to quickly distinguish between isolated renal injury and potentially more extensive intra-abdominal injury can contribute greatly to the expedient triage of trauma patients.

## Conclusions

While ultrasound is not the definitive diagnostic imaging modality in the setting of blunt trauma when CT is available, it is an invaluable tool for triage, particularly when assessing for intraabdominal injury. With the FAST accepted as an adjunct to the Advanced Trauma Life Support secondary survey and ultrasound education rapidly becoming a key component of medical training across disciplines, it is useful for providers to have a nuanced understanding of potential renal findings in the acute care setting.
